# Assessment of sustainable baits for passive fishing gears through automatic fish behavior recognition

**DOI:** 10.1038/s41598-024-63929-5

**Published:** 2024-06-07

**Authors:** Alexa Sugpatan Abangan, Kilian Bürgi, Sonia Méhault, Morgan Deroiné, Dorothée Kopp, Robin Faillettaz

**Affiliations:** 1 DECOD, L’Institut Agro, IFREMER, INRAE, 56100 Lorient, France; 2 Université Côte d’Azur, CNRS, ECOSEAS, Nice, France; 3 Institut Régional des Matériaux Avancés (IRMA), Ploemeur, France

**Keywords:** Behavioural ecology, Marine biology, Behavioural ecology, Environmental impact, Computer science

## Abstract

Low-impact fishing gear, such as fish pots, could help reduce human’s impact on coastal marine ecosystems in fisheries but catch rates remain low and the harvest of resources used for baiting increases their environmental cost. Using black seabreams (*Spondyliosoma cantharus*) as target species in the Bay of Biscay, we developed and assessed the efficiency of biodegradable biopolymer-based baits (hereafter bio-baits) made of cockles (*Cerastoderma edule*) and different biopolymer concentrations. Through a suite of deep and machine learning models, we automatized both the tracking and behavior classification of seabreams based on quantitative metrics describing fish motion. The models were used to predict the interest behavior of seabream towards the bait over 127 h of video. All behavior predictions categorized as *interested* to the bait were validated, highlighting that bio-baits have a much weaker attractive power than natural bait yet with higher activity after 4 h once natural baits have been consumed. We also show that even with imperfect tracking models, fine behavioral information can be robustly extracted from video footage through classical machine learning methods, dramatically lifting the constraints related to monitoring fish behavior. This work therefore offers new perspectives both for the improvement of bio-baits and automatic fish behavior recognition.

## Introduction

In our currently changing world, pot fisheries should not be considered “a return to the past, or to a lower technological level but rather a step towards a more sustainable future”^1^. Fish pots consist of two integral components: a bait to attract the target species and a containment unit. We here focus on the bait component by elaborating novel biodegradable, biopolymer-based baits (hereafter bio-baits) and developing a methodology to evaluate fish interest towards a bait in situ. Novel bait types can act as alternative to be more selective of the catch, to reduce cost, and to minimize its impact in the environment^2^. The landings of species harvested with pots, traps and crest, represent a small fraction of the total landings^3^ but these gears’ impacts are significantly lower than of active gears, both on the ecosystems^4^ and on the amount of greenhouse gas emissions throughout the harvest’s life cycle^5^. Pot fisheries therefore gain more attention as alternatives to towed fishing gears^4^ but innovative fish pots remain at their early stages and still require improvement to expand their usage in coastal fisheries.

Pots are highly diverse and different parts of a fish pot can be modified depending on the target species and local constraints (e.g., policy regulations, fishing practice, stock status)^1,6^. Floating pots with modified bait type help retain cod, *Gadus morhua*, while reducing bycatch of red king crab, *Paralithodes camtschaticus*^7^ while bottom-set pots target crustaceans while reducing bycatch of pelagic fish^8^. Pots have a lower operating cost and environmental cost than towed gears^1,5,9^ but designing efficient devices to target finfish is particularly challenging due to lower catch rates related to the quick consumption of baits compared to pots targeting crustaceans or cephalopods^6,10^.

Various species are currently harvested to obtain baits in order to attract target species. This harvest has been estimated to generate up to 31% of the greenhouse gas emissions generated throughout the life cycle of these passive gears fisheries^11,12^. In some extreme cases, the amount of fish harvest for baiting can even exceed the amount of target fish landed^13^. Thus, while passive gears are considered to be generally more sustainable than active gears, manufacturing efficient, eco-friendly bio-baits would enable to take out the harvest of edible species for baiting, further improving their sustainability. Long-lasting baits is among the most potent component for improving the currently limiting catch efficiency^6^. Biodegradable baits are still under development and have been previously elaborated with proteinaceous medium and water-soluble agents such as polyvinyl alcohol^14^, or by mixing natural and synthetic agents for longlines, showing encouraging results but only during laboratory trials^2,15^. Bio-baits based on biopolymers would enable to incorporate any amino-acid within the matrices, thus finding which pairs of matrices and amino-acid trigger attraction in target species would enable to obtain highly-selective baits. Their efficiency at attracting fish in situ has never been tested, while a target species behavior around a gear—or bait—has to be accounted for in each stage of design of a highly selective device with limited impact on non-target species^16–18^.

Even in controlled, laboratory set-ups, fish behavior can be difficult to predict^19^. This is even more striking in fishing gears environment where most information has been historically inferred from catch comparisons rather than from target species’ fine-scale behavior towards the device^19^, although recent studies now try to explicitly address species behavior (e.g. Ref.^20^). Behaviors are generally observed at a fine scale (< 10 m or < 10 min), focusing on how fish react to stimuli (attractive or repulsive) over a few seconds (e.g. Refs.^21,22^) to minutes of encounter (e.g. Refs.^23,24^) by scrutinizing swift changes in swimming direction, speed and even mouth or fin movements^6^. Direct observations of behaviors in fishing gears are thus becoming mandatory over long periods of time but at high spatiotemporal resolution^25^. These observations generate massive amount of data from which short behavioral sequences have to be extracted, which often dramatically reduces the amount of data actually analyzed for behavioral studies^26^, or with considerable human effort for manual processing or through citizen science projects^27^.

Computer vision coupled with deep learning models is now used for automatic object detection and tracking from underwater optics^28,29^, with a growing interest for automatizing behavioral data through objective quantification and synthesizing huge amounts of information in aquaculture to detect abnormal behavior and improve welfare^30^. Image quality and environmental factors (e.g., turbidity, luminosity, crowdedness) dictate the accuracy of such models^31,32^ but, in most cases, metrics can be derived from continuous tracking to provide insights into various aspects of fish behavior.

We here pursue a double objective: (1) develop long-lasting, biopolymer-based biodegradable baits to target black seabream and (2) assess the bio-bait’s efficiency in situ through a framework of artificial intelligence and machine-learning methods to automatically extract species tracks over a hundred hours of videos and process them through trajectory data-mining and movement ecology techniques developed in remote sensing and telemetry for planar data analysis^33–36^. This second aspect also aims at demonstrating how the automatization of fish behavior recognition will help in evaluating other types of baits than what we present here and species-specific fish-gear interactions.

## Methods

### Black seabream case study

In the Bay of Biscay, small-scale fisheries comprised 21% of the national production and black seabream accounted for 520 tons of landings in the area (2022; FOA Subarea VIII, Division A–D). Black seabream abundance peaks in the coastal area during the summer months, when catch of other species are relatively low. Currently, most landing originate from trawling and this seasonal fishery therefore presents an opportunity for the development of pots specifically designed to target this species^18^. Although there are past studies on the diet^37^, feeding locations^38^, and reproduction of black seabreams^39^ to improve fisheries management, little is known on their interactions with passive gears. Seabream pot development trials showed that a pot with a rectangular shaped entrance, transparent net, and floated circular form is the most optimal configuration to target black seabream^18^. The bait was chosen among 10 tested baits identified from the literature and interviews of recreational and professional fishers. Cockles, *Cerastoderma edule,* triggered the longest residence time of fish around the bait (about 2 h; ^40^). This bait was then used for the development of bio-baits.

### Developing bio-bait with a longer temporal span

#### Bio-baits composition and manufacturing

Three types of long-lasting bio-baits were developed and tested in situ: C17, C600, and Lactips. These bio-baits were produced from a mixture of water-soluble polymers biodegradable in a marine environment and powdered cockle (*Cerastoderma edule)* flesh (Supplementary Table S1). These materials were selected for their solubilization and biodegradable properties in cold water. Preliminary tests have shown different solubilization kinetics in both freshwater and seawater^40^. Lactips is a bio-sourced, natural polymer derived from animal proteins, soluble even in cold water and biodegradable in various environments, including the marine environment. The bio-baits C17 and C600 are synthetic polymers based on polyvinyl alcohol (PVOH). Preliminary biodegradation tests conducted in this study on these polymers have revealed that the C17 polymer biodegrades 10% slower than the C600 in a sand-seawater environment, after a 1-year incubation. The three types of biopolymers were chosen from off-the-shelf biodegradable plastic materials tested for their water-soluble properties. They showed a progressive release of particles suitable for in situ testing.

These polymers were combined with an attractant made of cockle flesh, which was chosen because it is easily accessible and used by local fishers while part of the seabream diet^37,41^. Cockles were dried in a vacuum oven, ground, and sieved to 300 µm. The cockle powder was then mixed with the water-soluble polymers in the molten state to facilitate the dispersion of the attractants in the plastic matrix, in an internal mixer (Brabender^®^ internal mixer; Supplementary Fig. S2a). The samples obtained were then granulated and thermo-compressed (Labtech^®^ LP-S-50 STD Press) to obtain 8 × 8 cm rectangular plates with a thickness of 3 mm (Supplementary Fig. S2b). The proportions of powder incorporated (30% cockle powder or dry weight of 100 g) were identical for the three bio-bait samples.

#### Bio-bait in situ attraction assessment

**Figure 1:**
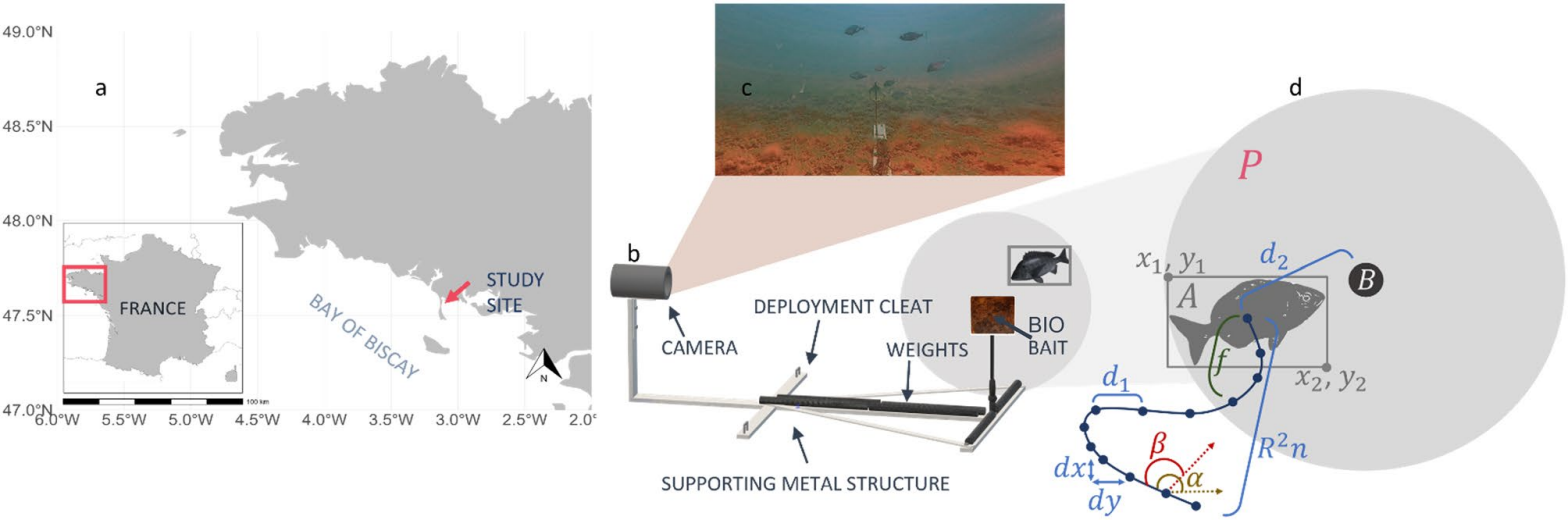
(**a**) Maps of the study site, generated in R v4.3.3 with sf v1.0.16. (**b**) Schematic of the underwater observation set-up, (**c**) Field of view of the camera with black seabreams swimming around from a pre-processed video frame, (**d**) Behavioral metrics quantification from a fish trajectory relative to stimuli where *B* = Bait point, *P* = Proximity-to-bait zone, *A* = annotation area, *d*_1_ = distance per fish move, *d*_2_ = distance to bait, *d*_*x*_ = change in x direction, *d*_*y*_ = change in y direction, *f* = frequency in proximity area, *R*^*2*^*n* = squared net displacement, α = absolute angle, β = relative angle to stimuli, *x*_1_ *y*_1_ = top left pixel coordinate of *A*, *x*_2_ *y*_2_ = bottom right pixel coordinate of *A*.

The study was conducted in the Bay of Quiberon (northern Bay of Biscay, France) over a 900 m^2^ rectangular experimental area (Fig. 1a) consisting of sand, mud, coarse sediments, and algae, with depths from 7 to 12 m depending on tides. In situ deployments took place between July 20th and 24th, 2020 when black seabream migrates to coastal areas during the summer. Each bio-bait was mounted in its own observation setup and deployed at the extremities of the study area, with placement times adjusted according to tidal conditions. The observation devices consisted of one GoPro® Hero (*4* or *7 Black*) camera mounted on a metallic frame weighted with ballasts (Fig. 1b), to maintain a fixed 2 m distance between the camera and the bait. The raw baits were attached to a nylon thread and tied to the metallic rod while the bio-bait plates were placed inside a mesh bag. Recordings were made with a 1920 × 1080 p resolution at a frame rate of 25 fps on a 128 Gb micro-SD. Each camera was connected to a 15,600 mAh battery, inside a waterproof housing, to allow for continuous 9-h recordings (constrained by the micro-SD capacity). Four observation devices were immersed each day, with one control to validate the presence of fish in the study area using raw cockle flesh and three with different types of bio-baits. To enable comparison of the bio-bait attractiveness, the four devices were deployed simultaneously before midday, for video footage to be recorded in daylight (see Fig. 1c for an example). Four trials were carried out per bait, over four days, generating a total of 127 h of raw footage, used to analyze fish behavior around the bait (Fig. 1d).

### Automatic fish behavior detection and quantification

#### Video processing

The dataset consists of 16 videos, lasting *circa* 9 h each. Only 15 videos are used for analysis due to a faulty camera on the control bait on day 1. Videos were subsampled at 5 fps (*ffmpeg* library^42^), a tradeoff between processing cost while retaining enough frames to capture swift fish movements^43^. All video processing (annotation, model training, testing, and validation) were performed with the image analytics platform VIAME^44^. Fish and bait classes were manually annotated frame-by-frame with bounding boxes with the standard COCO format (Common Objects in Context; ${x}_{1} {y}_{1}$, ${x}_{2}$, ${y}_{2}$). The trained detector used a Cascade Faster R-CNN as the framework for object detection^45^ coupled with Siamese network^46^ as the neural network architecture for object tracking. These architectures have shown robust performances in tracking fishes from underwater images as they can address occlusions^36,47,48^.

#### Groundtruth dataset for fish detector and tracker (DetTracker)

The groundtruth dataset used to train and test the DetTracker consisted of 54,425 annotations across 13,771 frames, comprising six classes focused on fish-bait interactions (“bio-bait”, “unknown fish”, “Labridae”, “plant”, “raw bait” and “*Spondyliosoma cantharus*”; Supplementary Fig. S3). There are a total of 46,923 annotations of fish (all species combined) and 6142 annotations of bait (bio-bait and raw bait combined). Classes were unbalanced but sufficient for robust bait detection given their homogeneity throughout the videos. For model training, annotations were gathered from cockle bait videos collected in 2019 with the same devices and protocol^18^, and which had already been partially annotated for seabream. This allowed us to train and evaluate a model on videos from a previous data collection and assess its performance at handling new data (the videos collected in the current study). For evaluating the DetTracker performance on the bio-bait videos from 2020, annotations were also gathered from random 300-frame sequences from the different bio-baits videos with various light and turbidity conditions to encompass most “fish events”, such as fish swimming in groups, individually, and behind the bait, for a total of 40 min of video validated. The model training and predictions were run on a GPU GeForce RTX3070i with 24 Gb of RAM.

#### DetTracker evaluation

To evaluate the detection accuracy, Average Precision (AP) and mean Average Precision (mAP) metrics were computed^49^. To evaluate tracking accuracy (i.e., ID-switch, cut tracks, etc.), the High-Order Tracking Accuracy metric (HOTA) was computed with TrackEval^50^. These model evaluations were used to evaluate which aspects of the localization, detection, and tracking are best handled and account for these performances when assessing model outputs. The trained model was then used to generate fish tracks in all 15 videos (i.e., over 2,624,000 frames). The tracker attributes the highest classification probability of an object among all instances throughout its track (e.g., when a fish swam alone while well visible, the probability of being a fish tends to be higher than during occlusions). Assuming that a single robust detection throughout a track is sufficient to attribute the predicted classification to the entire track, false positive fish tracks were removed by setting a 0.89 threshold on the maximum detection confidence, discarding most non-fish entities, such as floating plant debris. To ensure reliability, detections above 0.89 threshold underwent manual verification, confirming their identification as fish species.

#### Quantifying and classifying fish behavior (BeClassifier)

Fish behavior quantification and classification from annotated fish tracks (Supplementary Fig. S3) was achieved through a suite of quantitative metrics extraction and selection, clustering and Random Forest Classifier^51^ (Supplementary Fig. S4). To quantify and classify fish behavior, behavioral metrics were first calculated from the fish groundtruth to capture various aspects of fish movement and interaction with the bait, encompassing attributes such as proximity-to-bait, swimming, changes in direction, size, and orientation relative to the bait (Fig. 1d). A comprehensive pool of 104 behavioral metrics was considered (full list available in Supplementary Table S5) which was reduced through a step-wise process (Supplementary Fig. S4). Metrics that were highly correlated (Pearson’s r > 0.90) were compared to exclude redundant metrics since they tend to decrease the performance of Random Forest classifiers^51^ that were used for behavior classification. This resulted in a selection of 72 metrics.

To determine the number of behavior partitions, Gap statistic, Elbow and Silhouette method^52^ were applied on the 72 metrics, and identified that the optimal number of clusters to group the dataset is two (Supplementary Fig. S6). Two dichotomic fish behaviors were thus initially considered (*Interested* versus *Uninterested*, referred to as principal behavior) for the manual behavior annotation of the groundtruth dataset. A total of 301 fish were classified as either Interested or Uninterested and annotated separately by three trained annotators, who were then confronted to reach a consensus on all sequences.

Once the principal behavior dataset had been generated, the number of metrics required to classify them were further reduced to 12 metrics through a recursive elimination approach^53^. The selected variables were chosen from Random Forest runs with the lowest Out-of-bag (OOB) error rate and with the smallest set of features to account for processing cost. This was achieved through 1000 Random Forest iterations (default hyperparameters) averaged among all iterations, then plotted against model accuracy.

The dataset of groundtruth fish principal behavior along with the 12 most relevant metrics were then used to train and test the behavior classifier (hereafter BeClassifier). Annotations were pooled from manually annotated fish tracks from 2019 and 2020 datasets used for the DetTracker training. For each dataset, a stratified 50–50% split was done to ensure a balanced representation of fish tracks from the two behavior classes. This involves separately applying the split ratio to each behavior class within every dataset. The proportions were then aggregated to form the final dataset pool. However, we deliberately excluded interested fish from the bio-bait videos to test the BeClassifier ability to classify these fish without prior exposure. A total of 204 seabreams were used for training and 97 were used for testing. The split used for the different model training and testing are provided in (Supplementary Table S7).

The 12 metrics selected were then inputted to train a Random Forest model to classify fish behavior with fine-tuned hyperparameters (mtry = 1, ntree = 100, maximum nodes = 50, node size = 2, sample size = 50, class weighting with the inverse of class frequencies, and Gini as the split criterion) obtained by doing hyperparameter optimization with grid search^54^, as recommended for handling small datasets. The BeClassifier model was tested with a k-fold cross validation method to consider a diverse representation of Interested and Uninterested classes for each fold. Seabreams that were classified as Interested were all manually validated to evaluate the model’s performance at predicting interested fish, both on raw bait and bio-bait videos. Model evaluation was assessed using standard metrics (F1-score, accuracy, precision, and recall). The probability of a fish being interested was set to 0.52 based on the prevalence of incorrect classification below this threshold (i.e., when the probability of being Interested and Uninterested is near even).

#### Fine-scale behaviors classification

Interested fish exhibited behavior of exploration and different feeding movements where they either feed during a sustained period or bite and nibble the bait then quickly retreat, while Uninterested fish were either passing by or actively retreating away from the bait. We thus tested if accounting for these fine-scale behaviors improved the model’s prediction of the principal behavior and evaluated the output as for the two-class behavior classifier, then assessed the temporal distribution of these fine-scale behaviors around the bio-baits. Eight fine-scale behaviors (detailed in Supplementary Information S8) could be characterized based on the fish behavioral catalog from ^55^, showing different trajectory patterns (Supplementary Fig. S9). The same procedure was applied to the BeClassifier for the fine-scale behaviors classification, resulting in 30 relevant metrics and fine-tuned (mtry = 5, ntree = 650, maximum nodes = 50, node size = 5, sample size = 150, class weighting with the inverse of class frequencies, and Gini as the split criterion).

#### Data analysis

All data analysis was conducted in the R statistical software^56^, with the packages *tidyverse v2.0.0, dplyr v1.1.2* and *ggplot2 v3.4.2* for data management and graphics, *randomForest v4.7–1.1*, for the behavioral classifier, and *adehabitatLT v0.3.27* for behavioral metric calculation.

## Results and discussion

### Automatizing fish behavior classification from a large video database

#### Fish tracking in uncontrolled environment

Most studies aiming at developing low-impact, sustainable fish-targeting gear have been confronted with their low catch rates compared to traditional methods^6,10^. This case study is no exception as it is also confronted with a low number of observed fish. Still, the DetTracker identified a total of 202 fish tracks were detected above the species probability and behavior probability threshold throughout the 15 videos, with the model’s localization of fish instances presenting good performances at identifying seabream on the videos even with the overall low visibility (localization, Loc = 80.6%; detection, AP_seabream_ = 56.3%; Supplementary Fig. S10), consistent with previous studies^57,58^. Highly accurate trackers are developed on land^59,60^, and our results (tracking evaluation, HOTA_seabream_ = 23.99%) highlight how underwater imaging remains challenging. Trackers that are trained to follow regular motion and distinct features (e.g., SORT, DeepSORT, Siamese-based trackers) can perform poorly when tracking organisms that look alike and move erratically^61^. The fish present in the videos have almost uniform appearances, and their movements turn from being regular to erratic as they approach the bait. When tracking fish where movements are expected to be regular, such trackers can suffice^59,62,63^ but not with non-linear movements^61^; altogether, these factors explain the moderate model’s tracking accuracy, as is often the case in fish crowds^60,64^.

#### Predicting fish behavior from imperfect fish tracks

**Figure 2:**
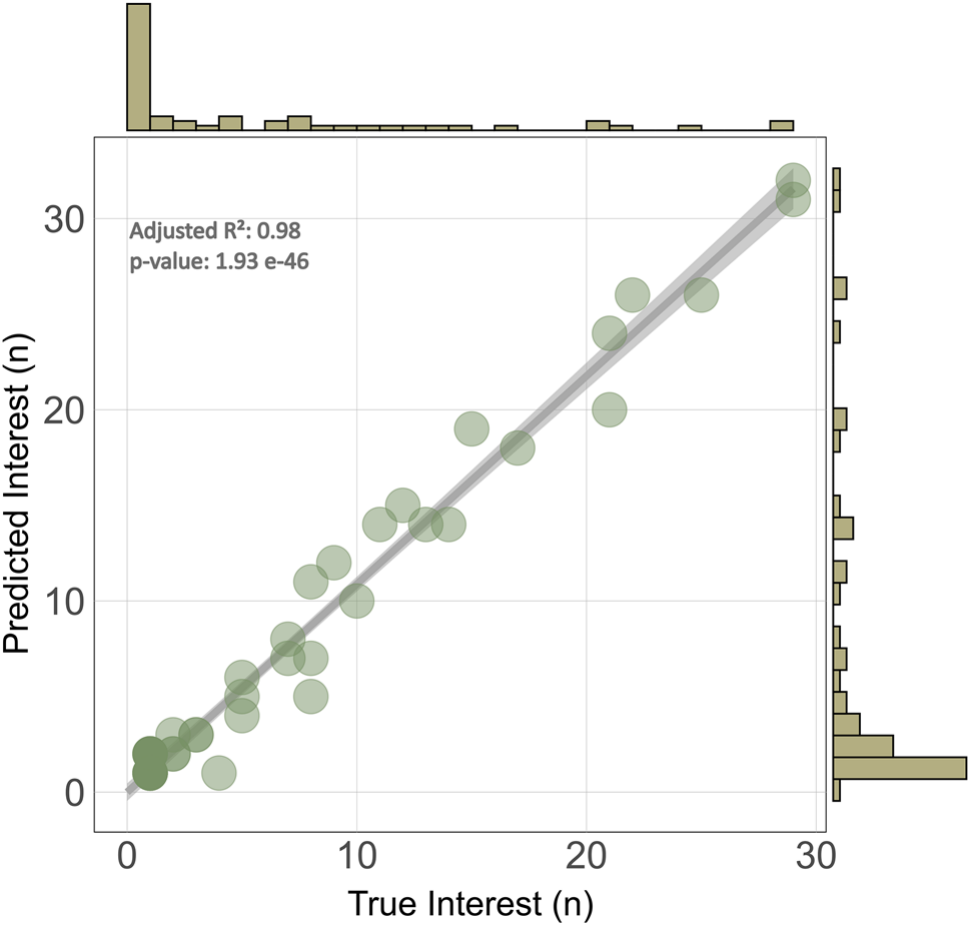
Predicted versus the groundtruth counts of interested fish, using the BeClassifier for different fish densities throughout the 127 h of video. The density of data points is shown at the top for the groundtruth (true interest) and on the right for the predictions from the BeClassifier.

To evaluate the bio-baits’ attraction power over long periods of time, the fish behavior around the bait needed to be identified among more than 2 million frames. The BeClassifier showed a high accuracy in classifying whether the fish is Interested or Uninterested from fish tracks (BeClassifier on principal behavior in the test set: Accuracy = 91.75%, F1 score = 90%, Precision = 90%, Recall = 90%; Supplementary Fig. S11). Validating the full video dataset further demonstrated a narrow linear relationship between the model's predictions and true interest, even in dense scenes (R^2^_adj_ = 0.98, *p*-value < 10^−10^; Fig. 2) and that the model was able to positively detect nearly all interested fish throughout the video (BeClassifier on principal behavior in the full dataset: Accuracy = 88.91%, F1 score = 94.13%, Precision = 90.72%, Recall = 97.80%). The inflated counts when three or less fish are present are results of split tracks of fish remaining for extended periods of time within the frame of the camera (Fig. 2; Predicted *Interest*). The moderate HOTA therefore did not propagate over the performance of automatic behavior predictions, and behavioral patterns were conserved even without exact start-to-end fish tracking.

In a static dataset, setting a high threshold to keep only the detections that are accurately predicted may suffice to highlight representative trends^65^. In extensive in situ recordings however, trackers may inevitably capture noisy fish tracks due to uncontrollable environmental conditions and fish movements^66^. Tracks can still show general trends when accumulated together^60^, and our study supports that “imperfect predictions” of fish movement still enable to draw accurate conclusions on spatial and temporal patterns in fish behavior, even with minimal human validation (40 min over 127 h of video, i.e. less than 0.04% of the final dataset) as long as error rate remains acceptable, or controlled for specific classes.

#### Coarse versus fine-scale behavior prediction

**Figure 3:**
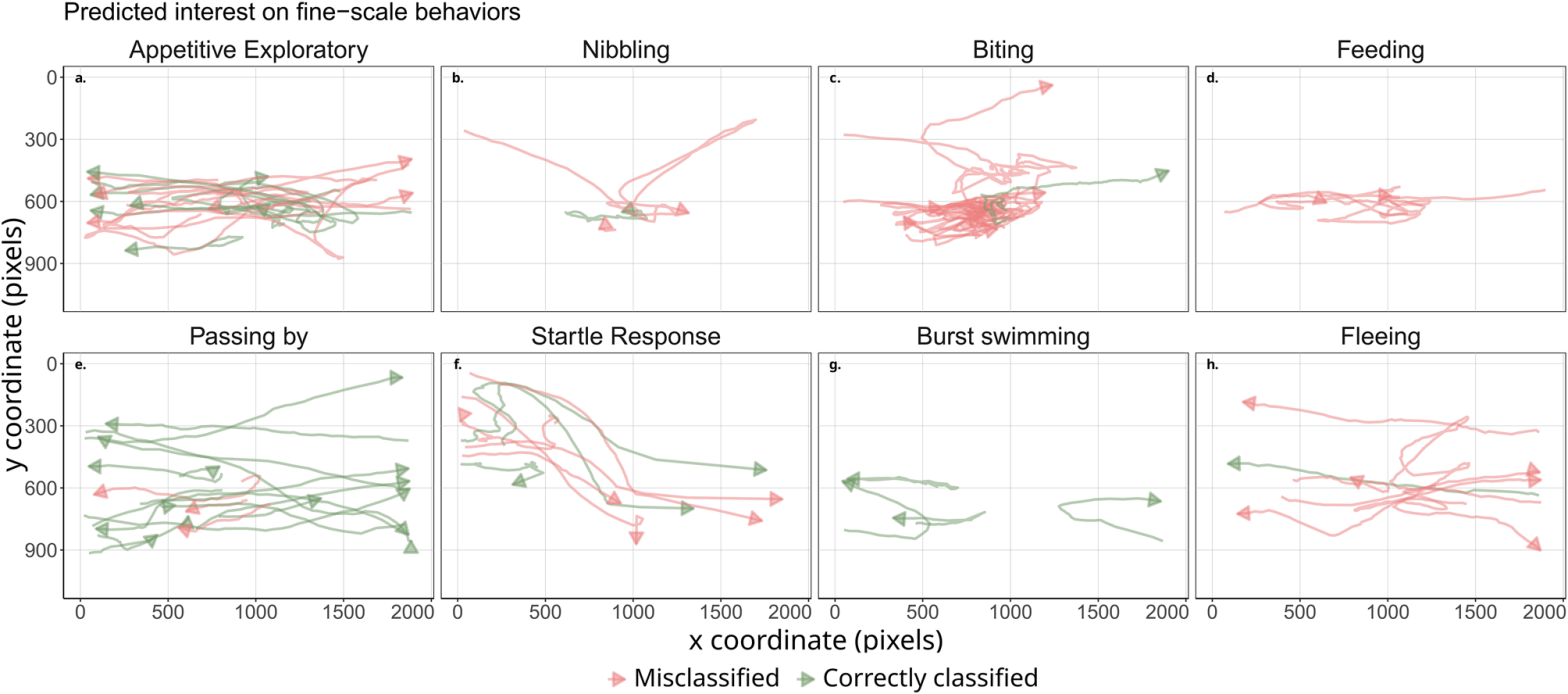
Fish tracks grouped into their corresponding true fine-scale behaviors colored by their predicted parent behavior (green tracks: correctly classified, red tracks: misclassified).

We here applied a cluster-then-classify approach, which highlighted two main behavioral groups from the fish trajectories (Supplementary Fig. S6 of gap statistics, silhouette and elbow results). Yet, categorizing fish behavior into two main, antagonist behavior remains a simplistic approach as its behavioral pattern changes over time, and variability if often more interesting than uniformity^67,68^. A fish can indeed show a strong interest toward the bait for short periods and then stay in the field of view for a longer duration without approaching the bait. Techniques for splitting animal movement paths have been developed to pinpoint the fundamental segments of motion within a trajectory^33^, and highlighted that animal interactions with the presence of stimuli are associated with a higher frequency of changes in movement. Nevertheless, the BeClassifier performed poorly at predicting the eight fine-scale behaviors (per behavior prediction statistics available in Supplementary Fig. S12 and Supplementary Table S13, and accounting for the fine-scale behaviors to predict parent behaviors (i.e., Interested for Feeding, Biting, Nibble and Exploring; Uninterested for Passing, Startle, Burst swimming and Fleeing; Accuracy = 84.48%, F1 score = 82.43%, Precision = 79.31%, Recall = 85.19%) did not enhance the model’s ability to discern the overarching patterns (Fig. 3). We thus argue that changes in movement over time can still be depicted by a one-behavior-per-track approach as long as the quantified behavior is related to fixed stimuli. To further improve accuracy, seabreams tracks may need to be segmented into fixed intervals to depict subtle changes in behavior, in particular over long tracks^69^. However, the objective of the study is to assess the overall interest of seabreams toward the bait where fewer behavior classes suffice.

#### The importance of objectives metrics and clusters definition for robust fish behavior recognition

The tracker’s shortcomings being superseded for behavioral quantification may also result from the objective cluster-then-classify approach used along with the behavioral metrics selection from a large (> 100) set of metrics (Supplementary Table S5). Defining behavior classes can be subjective^70,71^, and having a polarity or binary classification of positive and negative response to stimuli can serve as the principal behaviors to initially look for^72^. Here, the fish tracks were then described by objective metrics used to cluster behaviors, which were, in turn, associated with an explicit behavior (*i.e.*, the cluster-then-classify approach). Behavioral metrics with the highest ranking from the variable importance feature of Random Forest were all locomotion-based and stimuli-relative quantification of the fish’s swimming trajectory. Distance to bait and frequency in close proximity to stimuli are the strongest descriptors to classify behaviors around a cue as it is measured relative to the stimuli. Metrics like the number of fish present or the bounding box area as fish size proxy were uninformative. The cluster-then-classify approach thus enabled to handle the potential confusion of the trained behavior classifier when the fish’s interest manifested in different ways that could be attributed to opposite patterns (for instance, seabreams approaching the bio-bait through exploratory movements or quick approach and retreat; Fig. 3). As highlighted by the behavioral metric selection, locomotive-based metrics provide a more accurate way to identify behavior than relying solely on responses to stimuli and personality traits^73,74^.

### Bio-bait efficiency in situ assessment

**Figure 4:**
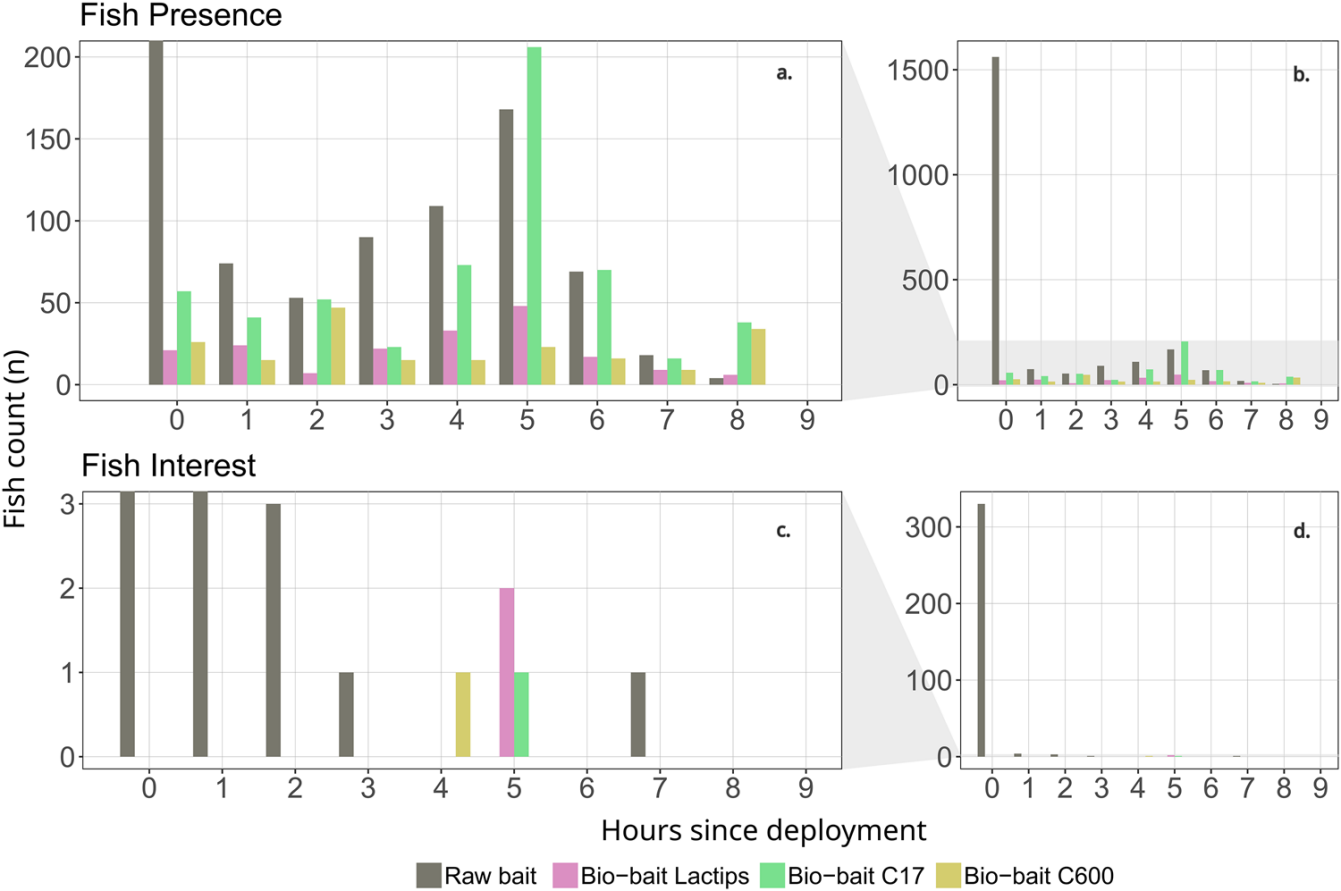
Seabream presence on the footage and interest toward the baits along the 9-h deployments. (**a**) Count of fish activity in bio-baits based on model predictions, (**b**) Full-scale showing all counts in raw bait, (**c**) True interest of fish in bio-baits based on actual observations, (**d**) Full-scale showing all interest in raw bait.

The presence and behavior of seabreams were then detected throughout the database to determine the efficiency of the bio-baits, following the assumption that fish interest would increase over time for bio-baits and decrease for raw bait. The count of fish activity is based on model predictions (Fig. 4a,b), while the fish interest is based on actual observations (Fig. 4c,d) since all model predictions for interest behavior have been manually validated. Seabreams were observed every day in the experimental area, and raw cockle bait attracted more fish than bio-baits (Kruskal–Wallis: *chi*^*2*^ = 12.021, *p* = 0.00731; Dunn test on raw bait vs bio-baits: all *p* < 0.05; Fig. 4a,b). Yet, peak activity around raw bait was limited to the first hour of immersion and interest afterwards were solely directed towards the nylon, which hold the raw bait until consumed, or the rod. Some fish detected on the raw bait videos during the second half of the deployment, with the bait already consumed, were mostly curious towards the apparatus and camera. Seabream interest toward the bio-baits was detected during the 4th and 5th deployment hours (Fig. 4c,d) where they showed feeding, nibbling and biting behaviors towards the bio-baits. Other species such as the European seabass (*Dicentrarchus labrax*) also approached and circled around the bio-baits during the 5th hour. Raw bait therefore outperforms bio-baits in terms of overall activity, but bio-baits show potential in terms of longevity as they continued to lure fish several hours post deployment and were also consumed by the target species.

### Bio-bait and its future implications

The duration of baited fish pot deployment is limited by the bait’s efficiency to still lure fish over an extended period of time, which are costly to regularly replace and re-deploy^75^. Our results do highlight some potential for bio-baits to trigger fish interest several hours after raw bait has been consumed and attracted finfish only with no crustaceans spotted wangling around bio-baits as opposed to raw cockle baits. All bio-baits exhibited an activity peak in between the 4th and 6th hour after deployment while raw baits’ peak interest occurred within the first hour only (Fig. 4). Raw bait still had a much stronger attractiveness during this period since it is more potent and has a wider spread of odor diffusion from natural prey cues the species may be used to^76^. The results also show that only the bio-bait continued to attract fish several hours after being deployed. In a fisheries perspective, combining the raw and bio-baits would maintain the strong initial attractiveness while increasing odor diffusion over time. A natural bait, having a faster odor diffusion, would initiate the chemical cue, followed by the artificial bait that would gradually release odors after a few hours of immersion, but this would require obtaining natural baits from other sources and may, in turn, reduce the overall sustainability of the fisheries. Economic constraints should also be considered given that, as of now, the manufacturing cost of an artificial bait is higher than that of a natural bait. This may be compensated by reduced cost for professional's maintenance and sourcing of natural baits^12^. Several initiatives aim to value fisheries discards such as fish head, viscera and tail for baiting^2^ extracting their amino-acids and incorporating them into bio-bait matrices could be a solution but, again, only if discards are unavoidable.

This study remains exploratory, and several points of improvement remain to be addressed, in particular on the bait’s attractiveness. Even if preliminary experiments had been carried out, formulation optimization would be necessary to improve control bait release and attractiveness, for example by modifying the percentage of attractant in the mixture. While prior studies highlighted the significance of amino acids in water-soluble extracts in activating feeding behavior in fish, other factors like visual cues and the palatability of the bait itself play significant roles in fish responses^2,19,77,78^. However, synthetic amino acid mixtures may be enticing but seldom match the effectiveness of natural food extracts^76^. Previous research has identified black seabreams as an omnivorous and opportunistic species, consuming a diverse diet including mysidacea, crustaceans, polychaetes, and algae^41,79^. Improving long-lasting baits based on biopolymers may therefore not only consider potency but its composition in terms of amino acids. While here only cockles’ amino acids were tested, others could have triggered a stronger response but are yet to be identified. The appearance of the bio-baits can also be improved by having prey-shaped forms with subtly colored appearance^78^. One of the advantages of plastic materials is their ease of processing. Specific gynoid-type structures, for example, which can be envisaged using 3D printing technology, could improve the release of attractants, while working on the bait's shape to optimize visual appeal. The composition of the bio-bait could thus still be largely improved to diffuse more potent odors along with providing a more desirable texture.

Other amino acids and bio-bait shapes should still be assessed to increase its attractiveness. Bio-bait has the potential to be more sustainable than raw bait as the amount of organic material incorporated in the mixture can be controlled, and thus reduced. Still, it is important to consider how the decrease in organic material affects the mixture process, as well as whether this reduction weakens the distinctive cockle scent, potentially making the baits less appealing to fish. Using cockles from food waste could valorize leftovers that would otherwise end up in landfills. Various types of synthetic artificial baits have been developed that have explored various bait mixtures. Bait with a protein-rich gel interior and an insoluble skin-like exterior, incorporating fish waste to emit natural odors, have been introduced decades ago^14^. Innovations followed that utilized water-soluble polymers and plasticizers mixed with fish extracts, such as the biopolymer used in this study, to allow for controlled dispersion. Synthetic bait for crustacean capture that releases attractants at a controlled rate, showed effectiveness with various crab species and American lobsters in field trials^80^. These developments in bait technology aim to minimize the use of natural forage fish. When evaluating the viability and acceptance of these bio-baits by fishers, it is thus crucial to think about the source and quantity of cockles used and their impact on the environment. Here, the preliminary study on the biodegradation of the bio-baits used in this study found that after 180 days, the degradation rate reached 50–60%. However, the rate slowed considerably thereafter, stabilizing between 80 and 90% after 600 days in a controlled marine environment. This suggests a significant but gradual breakdown of the baits over time, which would, over longer time scale, strongly reduce the number of natural resources used for baiting.

Furthermore, for an exploratory study like this one, site selection was conducted randomly. In future trials, deploying the baits should also be done by varying locations while maximizing the distance among the sites^81^. This would enable to minimize bias from the same fish exploring the bait at different sites, or over repeated days. The fish activity may also be refined by monitoring the behavior of fish outside the field of view of the camera pointing at the bait, to estimate the optimal time needed to consider a fish entering the field of view as a new—versus a repeated—observation of fish (e.g., Refs.^82,83^).

This study presents new polymer-based and cockle powder bio-baits to attract black seabream over a longer period than classical baits. While the bio-baits efficiency remained low, it showed potential for bio-baits to attract fish after the natural bait has been consumed. To analyze the data, we developed a semi-automatic method to detect, track, and classify seabream behaviors from videos. We have shown that reliable behavior quantification can be achieved when applied to large volumes of data such as continuous video recordings and marginal validation, even with noisy patterns varying conditions on the in situ recording. We emphasize the ability of trackers, that may be deemed to have poor performance, to still have a significant importance in track-based behavior classification. Our approach, therefore, goes further than previous studies in terms of automatic fish behavior classification from tracks over long periods in situ (e.g., Ref.^66^), and widens our range of observational capabilities in assessing fish—or other aquatic organisms—behavior, interactions or response to stimuli in uncontrolled environment. From a fishing technology perspective, the fine knowledge of species behavior would enable conceiving highly selective, effective and sustainable, fishing gears and practices.

### Supplementary Information

Supplementary Information.

## Data Availability

The data and codes are available upon request addressed to the corresponding author, R.F., robin.faillettaz@ifremer.fr, or A.S.A., abanganalexa@gmail.com.
