# Supplementary material for: Assessment of sustainable baits for passive fishing gears through automatic fish behavior recognition
Source: Sci Rep. 2024 Jun 7;14:13110. doi: 10.1038/s41598-024-63929-5 (PMC11161462; doi:10.1038/s41598-024-63929-5)
Supplement: Supplementary file 1 — Supplementary Information. [file 41598_2024_63929_MOESM1_ESM.pdf]

SUPPLEMENTARY INFORMATION

Assessment of sustainable baits for passive fishing gears through automatic fish behavior recognition

Alexa Abangan[1], Kilian Bürgi[1,2], Sonia Méhault[1], Morgan Deroiné[3], Dorothée Kopp[1], Robin Faillettaz[1*]

[1]DECOD, L'Institut Agro, IFREMER, INRAE, 56100, Lorient, France
[2]Université Côte d'Azur, CNRS, ECOSEAS, Nice, France
[3]Institut Régional des Matériaux Avancés (IRMA), Ploemeur, France

[*]Correspondence to: robin.faillettaz@ifremer.fr

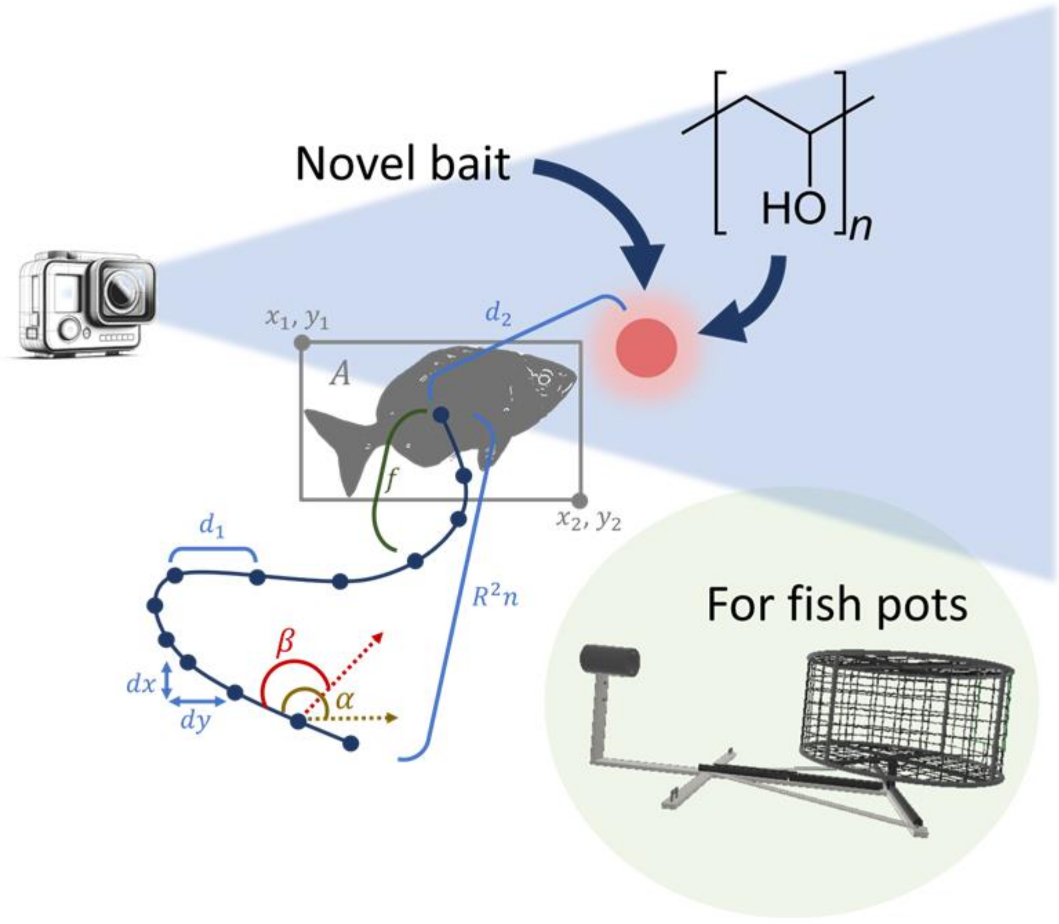

Supplementary Table S1: *Technical specifications of the bio-bait.*

Bait	Polymers	Producers	Brand	Grade	Diffusion time	Melt Flow Index (g/10 min)	Melting Temperature
**C17**	Polyvinyl alcohol (PVOH)	Kuraray	MowiFlex™	C600	Slow diffusion (50% diffusion over 24 days)	14-20	170°C
**C600**	Polyvinyl alcohol (PVOH)	Kuraray	MowiFlex™	C17	Slightly faster diffusion (60% mean diffusion over 24 days)	15-35	165°C
**Lactips**	Polyvinyl alcohol (PVOH) obtained from 60% milk casein proteins	Lactips	CareTips®	CareTips 300D	Faster diffusion than C17 and C600	Undetermined	110°C

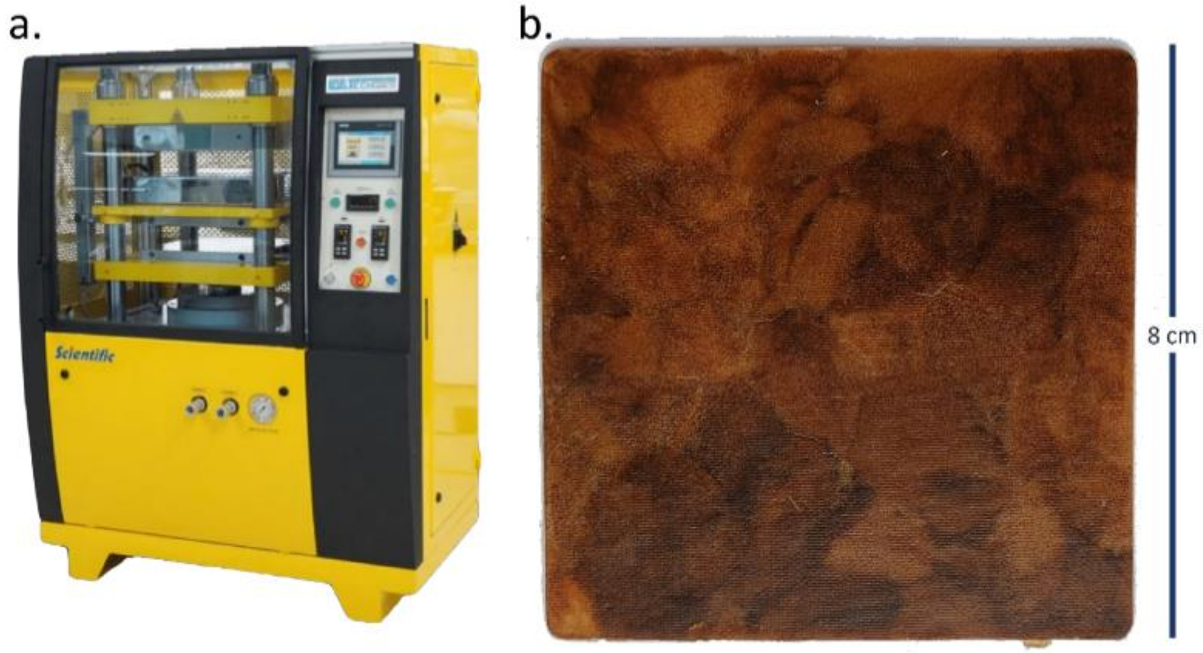

Supplementary Figure S2: *a) Brabender® internal mixture machine, and b) Example of the bio-bait sample in its final form.*

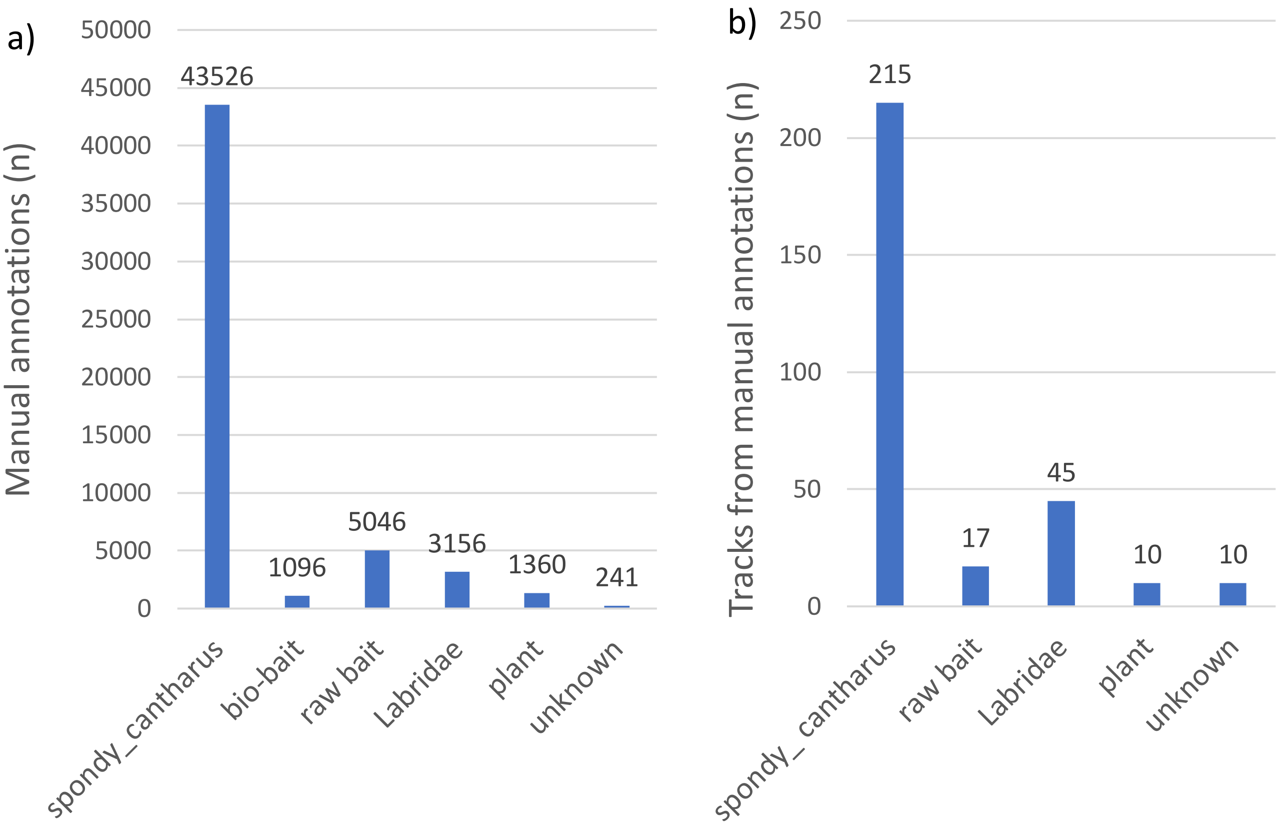

Supplementary Figure S3: *Groundtruth/manual annotations (a) and tracks (b) for the different annotation labels used for model training and testing.*

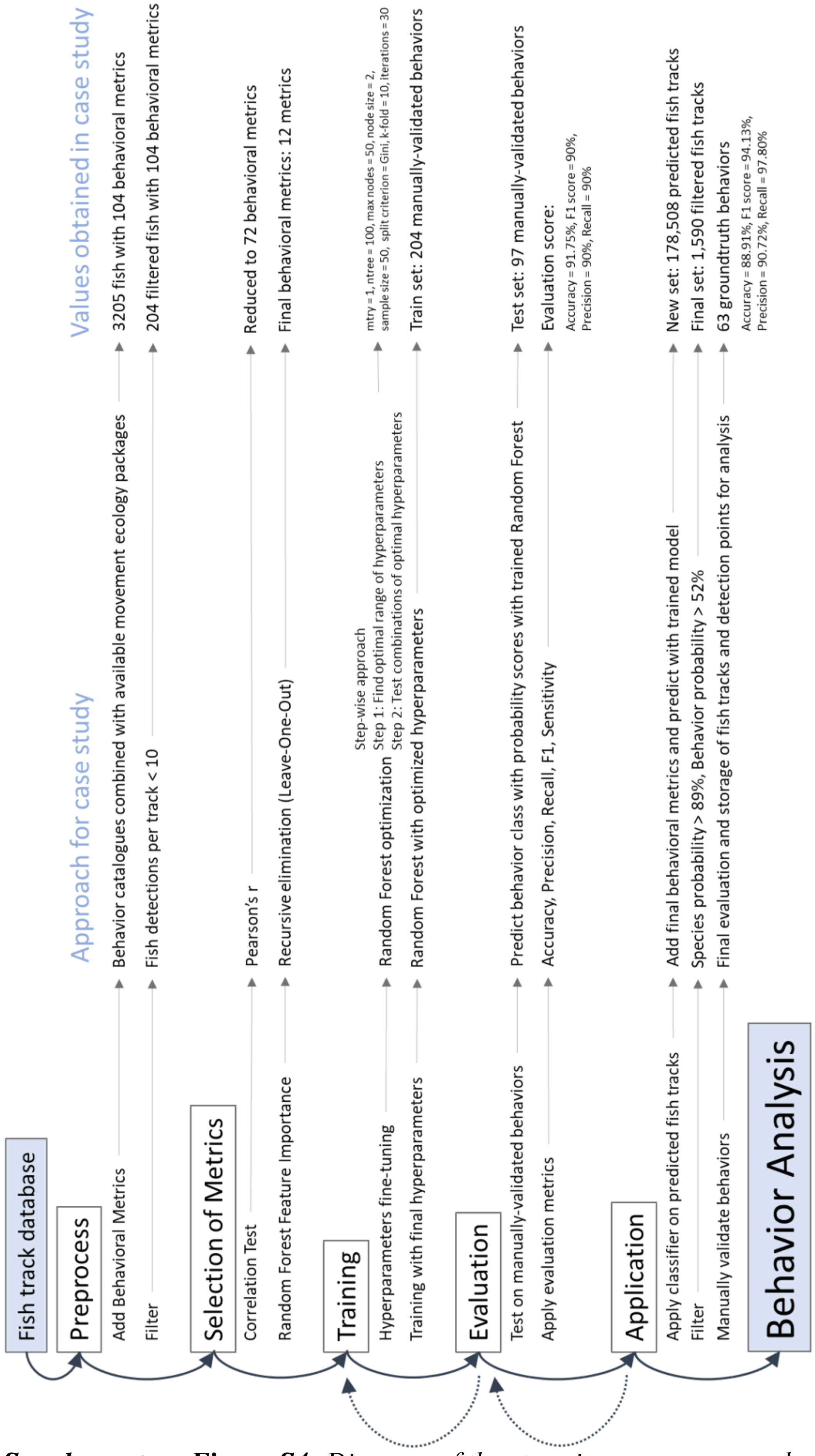

Supplementary Figure S4: *Diagram of the stepwise process towards an automatic behavior classification.*

Supplementary Table S5: *Exhaustive list of behavior metrics considered.*

Morphological Metrics

	Behavioral Metrics	Mathematical Representation	Description
1	Fish size (mean)	$mean_A = \frac{1}{n}\sum_{i=1}^{n} A_i$<br>Where:<br>• $mean_A$ is the mean average of fish size<br>• $\sum_{i=1}^{n} A_i$ is the sum of all individual fish sizes from A_1 to A_i	Area of the fish annotation for each of its successive move. Larger annotations can indicate the fish is closer to the camera. A rapid increase could suggest movement toward the bait, while a rapid decrease could indicate retreat.
2	Fish size (median)	$median(A) = A_{(\frac{n+1}{2})}$<br>$median(A) = \frac{A_{(\frac{n}{2})} + A_{(\frac{n}{2}+1)}}{2}$	
3	Fish size variability (standard deviation)	$\sigma\ (A_{annotation})$	
4	Fish size variability (interquartile range)	$IQR(A_{annotation})$	
5	Fish size change rate (mean)	$mean\left(\frac{\Delta A}{\Delta time}\right)$	
6	Fish size change rate (median)	$median\left(\frac{\Delta A}{\Delta time}\right)$	
7	Minimum fish size	$min(A_{annotation})$	
8	Maximum fish size	$max(A_{annotation})$	
9	Fish size (25th percentile)	$Q_1(A) = F^{-1}(0.25)$<br>Where:<br>• $Q_1(A)$ is the 25th percentile of fish size (A)<br>• F^{-1} is the inverse of the cumulative distribution function (CDF) of A	
10	Fish size (75th percentile)	$Q_3(A) = F^{-1}(0.75)$<br>Where:<br>• $Q_3(A)$ is the 75th percentile of fish size (A)<br>• F^{-1} is the inverse of the cumulative distribution function (CDF) of A	

Positional Metrics

	Behavioral Metrics	Mathematical Formula	Description
11	Relative size-to-distance ratio (mean)	$mean(\frac{distance_{move}}{time})$	Fish size over the distance of the fish's successive moves relative to the bait. An approximation considering the 2D limitation into the fish's three-dimensional movement relative to the bait.
12	Relative size-to-distance ratio (median)		
13	Distance to bait (mean)	$mean(d_{fish \to stimuli})$	Distance of the center points of the fish (x, y) to the center point of the bait (x, y). Short distances can indicate interest.
14	Distance to bait (median)	$median(d_{fish \to stimuli})$	
15	Minimum distance to bait	$min(d_{fish \to stimuli})$	
16	Maximum distance to bait	$max(d_{fish \to stimuli})$	
17	Distance to bait (25th percentile)	$Q_1(d_{fish \to stimuli}) = F^{-1}(0.25)$	

18	Distance to bait (75th percentile)	$Q_3(d_{fish\rightarrow stimuli}) = F^{-1}(0.75)$	
19	Distance to bait variability (standard deviation)	$sd(d_{fish\rightarrow stimuli})$	
20	Distance to bait variability (interquartile range)	$IQR(d_{fish\rightarrow stimuli})$	
21	Distance to bait skewness	$skewness(d_{fish\rightarrow stimuli})$	
22	Kmeans-based proximity zones	$kmeans(d_{fish\rightarrow stimuli}), centers = 3$	Spatial distribution of a fish (Close, Mid, Far) relative to the bait based on k-means clustering
23	Frequency in most visited zone	f_{mode}	
24	Entropy of fish swimming movement	$P = \frac{frequency}{total\ count}$ $E = -\sum(P\ x\ log_2 P)$	Measure of the randomness of movement across the different proximity zones
25	Frequency of entering the close proximity zone	$f_{entering}$	Frequency of how fish go back and forth from the bait that could indicate hesitation
26	Total frequency in close proximity zone	f_{close}	Time spent close to the bait
27	Total frequency of fish key points (head and tail) in close proximity zone	$f_{closespread}$	Time spent close to the bait including the key points of fish's head and tail
28	Number of fish per frame (mean)	$mean(\frac{N_{fishes}}{N_{frame}})$	Average number of fish in a frame
29	Number of fish per frame (median)	$median(\frac{N_{fishes}}{N_{frame}})$	
30	Number of fish per frame (standard deviation)	$sd(\frac{N_{fishes}}{N_{frame}})$	
31	Number of fish per frame (interquartile range)	$IQR(\frac{N_{fishes}}{N_{frame}})$	
32	Number of fish per frame (25th percentile)	$Q_1\left(\frac{N_{fishes}}{N_{frame}}\right) = F^{-1}(0.25)$	
33	Number of fish per frame (75th percentile)	$Q_3\left(\frac{N_{fishes}}{N_{frame}}\right) = F^{-1}(0.75)$	
34	Minimum number of fish per frame	$min(\frac{N_{fishes}}{N_{frame}})$	
35	Maximum number of fish per frame	$max(\frac{N_{fishes}}{N_{frame}})$	
36	Frequency of directional change relative to bait	$f_{directional}$	Measure of how the fish meanders or move purposefully around the bait
37	Directional change variability	$sd(f_{directional})$	
38	Relative angle to bait (mean)	$mean(\beta)$	Angle of fish trajectory relative to the bait
39	Relative angle to bait (median)	$median(\beta)$	
40	Relative angle to bait variability (standard deviation)	$sd(\beta)$	

41	Relative angle to bait variability (interquartile range)	$IQR(\beta)$	
42	Relative angle to bait (25th percentile)	$Q_1(\beta) = F^{-1}(0.25)$	
43	Relative angle to bait (75th percentile)	$Q_3(\beta) = F^{-1}(0.75)$	
44	Maximum relative angle to bait	$max(\beta)$	
45	Weighted mean x point	$\bar{x}_{weighted}$	Change in x weighted by the bait position
46	Weighted mean y point	$\bar{y}_{weighted}$	Change in x weighted by the bait position
47	Mean bearing	$\bar{\theta}_{bearing}$	average directional angle of the fish for each successive move referenced to the North (y axis)
48	Rayleigh statistic	$R_{rayleigh}$	Parametric measure of uniformity of the distribution of the bearing angles of a fish
49	Q1	Q_1	Sum of the cosines of the bearing angle
50	Q2	Q_2	Sum of the sines of the bearing angle
51	K1	K_1	concentration parameter of a von Mises distribution (analogous to the normal distribution for circular data)
52	K2	K_2	concentration parameter of a von Mises distribution (analogous to the normal distribution for circular data)
53	Proportion of q1q2	$\frac{Q_1}{Q_2}$	Mean direction of the bearing angle
54	Likelihood	$\mathcal{L}$	Measure of how well the data fits a bimodal distribution
55	RAO	R_{rao}	Non-parametric measure of uniformity of the distribution of the bearing angles of a fish

Motion Metrics

	Behavioral Metrics	Mathematical Formula	Description
56	Speed (mean)	$mean(d/s)$	Planar distance travelled by the fish per frame
57	Speed (median)	$median(d/s)$	
58	Speed variability (standard deviation)	$sd(d/s)$	

59	Speed variability (interquartile range)	$IQR(d/s)$	
60	Acceleration (mean)	$mean(\Delta d/s)$	Change in distance travelled by the fish per frame
61	Acceleration (median)	$median(\Delta d/s)$	
62	Acceleration peak	$max(\Delta d/s)$	
63	Distance per move (mean)	$mean(d)$	Euclidean distance of each successive move from one point to another
64	Distance per move (median)	$median(d)$	
65	Distance per move variability (standard deviation)	$sd(d)$	
66	Distance per move variability (interquartile range)	$IQR(d)$	
67	Distance per move (25th percentile)	$Q_1(d) = F^{-1}(0.25)$	
68	Distance per move (75th percentile)	$Q_2(d) = F^{-1}(0.75)$	
69	Minimum distance per move	$min(d)$	
70	Maximum distance per move	$max(d)$	
71	Absolute angle (mean)	$mean(\alpha)$	Angle of fish trajectory relative to a fixed direction (North – navigation)
72	Absolute angle (median)		
73	Absolute angle variability (standard deviation)	$median(\alpha)$	
74	Absolute angle (interquartile range)	$IQR(\alpha)$	
75	Absolute angle (25th percentile)	$Q_1(d) = F^{-1}(0.25)$	
76	Absolute angle (75th percentile)	$Q_2(d) = F^{-1}(0.75)$	
77	Minimum absolute angle	$min(\alpha)$	
78	Maximum absolute angle	$max(\alpha)$	
79	Squared net displacement (mean)	$mean(R^2n)$	Overall change in position of an animal, calculated as the shortest distance from the starting point to the end point of its path, regardless of the path taken
80	Squared net displacement (median)	$median(R^2n)$	
81	Squared net displacement variability (standard deviation)	$sd(R^2n)$	
82	Squared net displacement (interquartile range)	$IQR(R^2n)$	Lowest to highest displacement
83	Squared net displacement (25th percentile)	$Q_1\left(R^2n\right) = F^{-1}(0.25)$	

84	Squared net displacement (75th percentile)	$Q_1\left(R^2n\right) = F^{-1}(0.25)$	
85	Maximum squared net displacement	$max(R^2n)$	
86	Total squared net displacement	$Total\ R^2n$	
87	Change in x direction (mean)	$mean(\Delta x)$	Difference in the horizontal (often East-West) position of an animal between each successive move
88	Change in x direction	$median(\Delta x)$	
89	Change in x direction	$IQR(\Delta x)$	
90	Change in x direction	$sd(\Delta x)$	
91	Change in x direction	$Q_1(\Delta x) = F^{-1}(0.25)$	
92	Change in x direction	$Q_1(\Delta x) = F^{-1}(0.25)$	
93	Change in x direction	$min(\Delta x)$	
94	Change in x direction	$max(\Delta x)$	
95	Change in y direction	$mean(\Delta y)$	
96	Change in y direction	$median(\Delta y)$	
97	Change in y direction	$IQR(\Delta y)$	
98	Change in y direction	$sd(\Delta y)$	
99	Change in y direction	$Q_1(\Delta y) = F^{-1}(0.25)$	
100	Change in y direction	$Q_1(\Delta y) = F^{-1}(0.25)$	
101	Change in y direction	$min(\Delta y)$	
102	Change in y direction	$max(\Delta y)$	
103	Mean x point	$mean(x_{center})$	Change in x and y multiplied with distance to bait
104	Mean y point	$mean(y_{center})$	

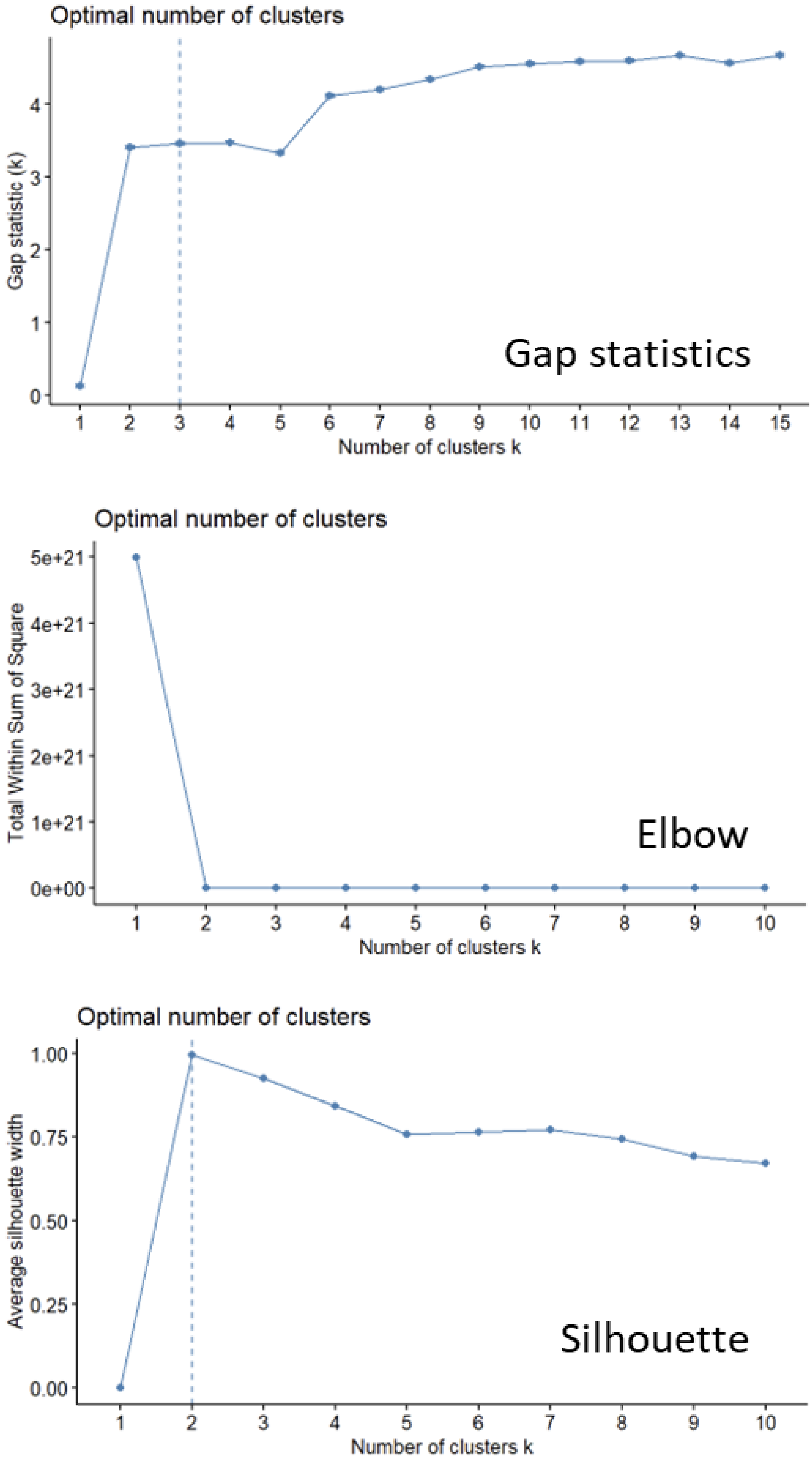

Supplementary Figure S6: *Gap statistics, Elbow and Silhouette method to determine number of behavioral groups.*

Supplementary Table S7: *Stratified 50-50 split of fish count for training (green) and testing (orange).*

**Datasets**	***Interested***		***Uninterested***	
**Raw bait 2019**	82		122	
**Raw bait 2020**	16	16	15	16
**Bio-bait 2020**	19		21	21

Supplementary Information S8: *The behavior catalog established in this study for* Spondyliosoma cantharus.

For ***Interested*** behaviors: ***Appetitive exploratory*** occurred for decreasing rate of swimming and distance traveled around the bait with frequent directional changes (frequency of directional change = 18 moves). ***Feeding attempt*** is characterized by prolonged presence (on average 95 frames) with a close proximity to the bait (< 21 pixels). ***Biting*** is when fish actively bite the bait with quick movement toward the bait and physical contact. ***Nibbling*** occurs when fish make smooth contact with the bait as part of object exploration.

For ***Uninterested*** behaviors: ***Fleeing*** is characterized by accelerating movement away from other fish or the bait. ***Passing*** *by* is when fish undertook escape-like turns, characterized by fast, large-angle turns that involve bending of the entire body with high angular velocity. ***Burst*** *swimming* was observed on fast forward swim fish with large bend angles, large distance per move and greater yaw that during slow swimming. ***Startle response*** corresponds to the fastest escape response, starting with C-shape body position followed by a rapid, straight move away from its current position.

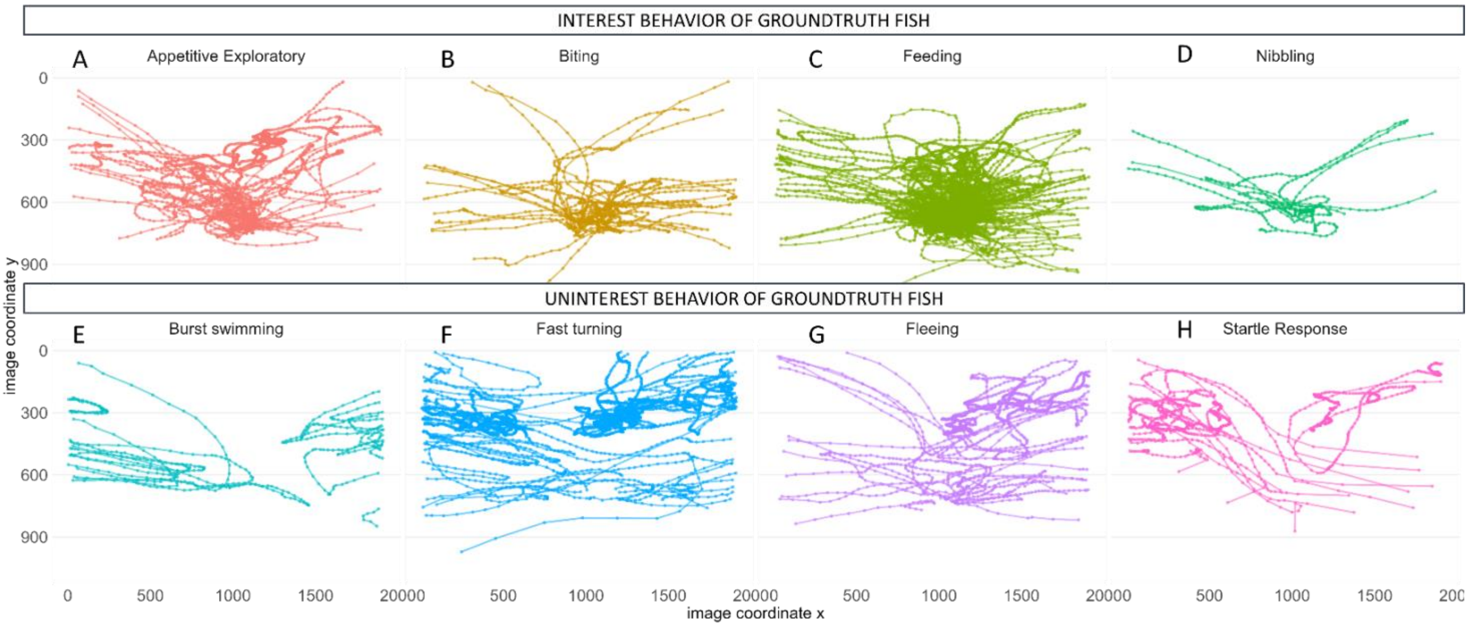

Supplementary Figure S9: *Fine-scale behavior trajectories of Spondyliosoma cantharus, extracted from the videos of 2019.*

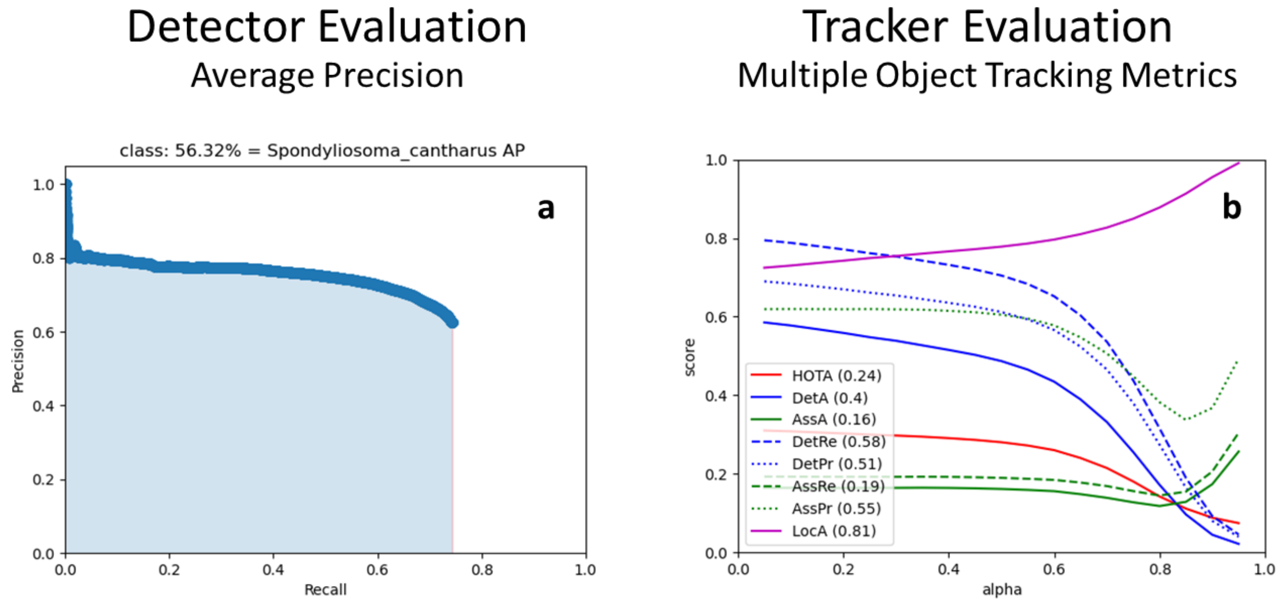

Supplementary Figure S10: *DetTracker models evaluation, for the a) mean average precsion (mAP) of the fish detector and b) the tracker using trackEval, available at https://github.com/JonathonLuiten/TrackEval.*

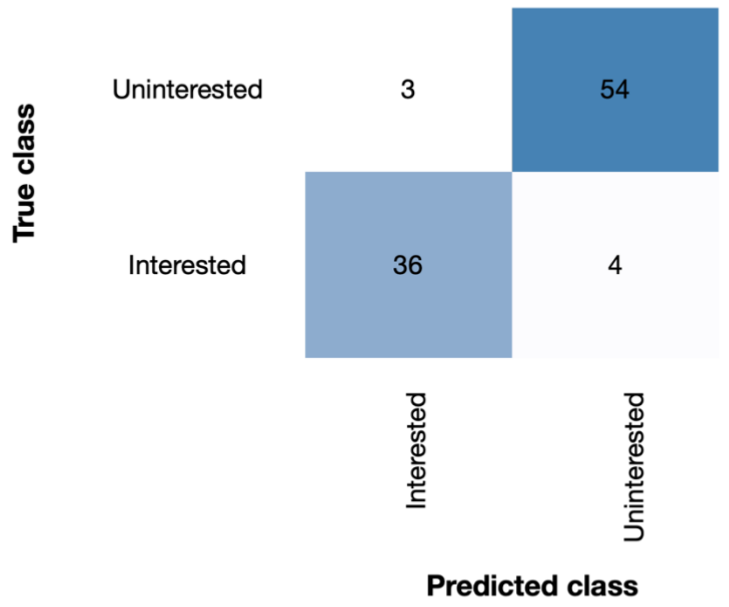

Supplementary Figure S11: *BeClassifier confusion matrix on the two principal behaviors from the groundtruthed bio-bait videos.*

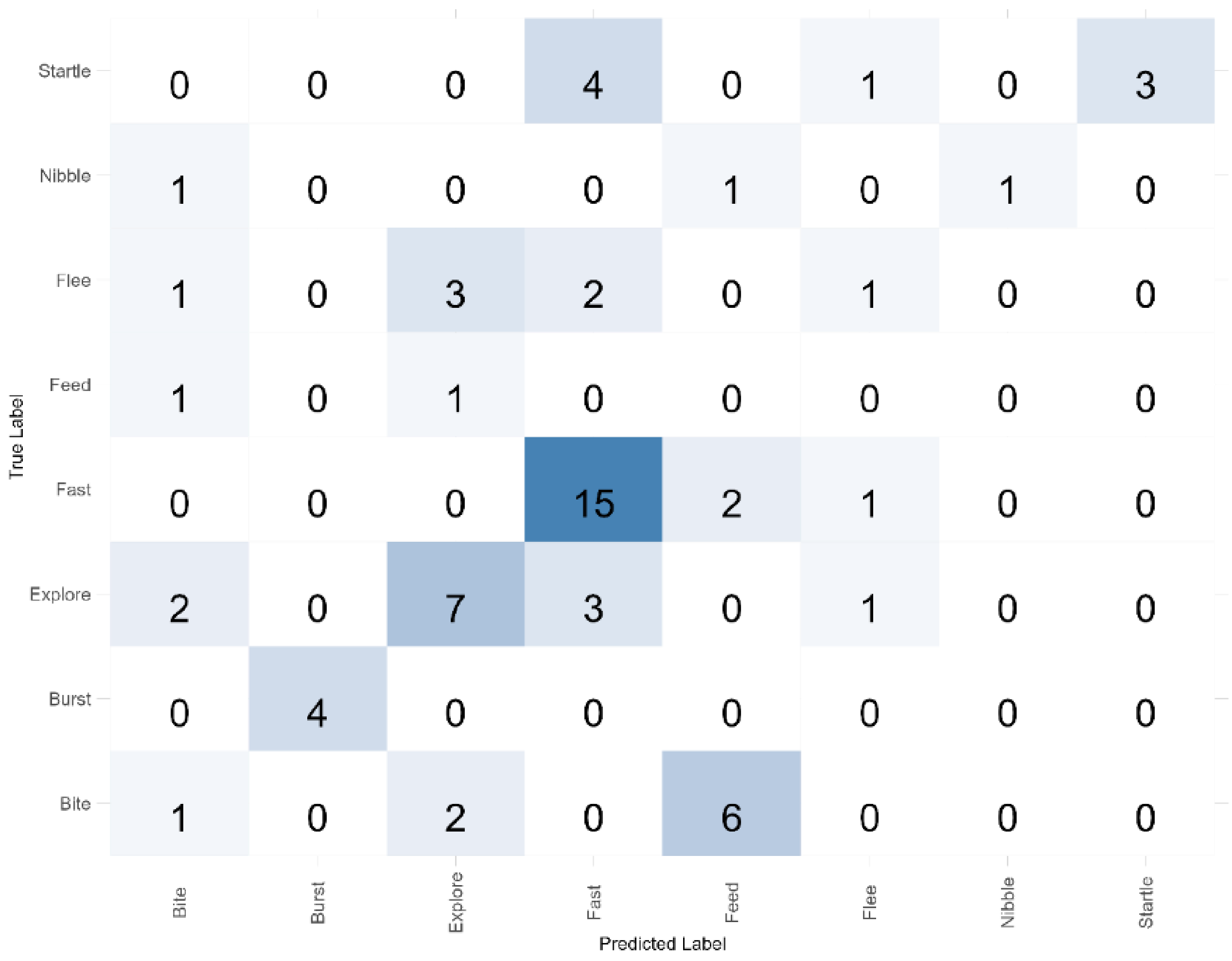

Supplementary Figure S12: *BeClassifier confusion matrix on the eight fine-scale behaviors from the full test set.*

Supplementary Table S13: *BeClassifier evaluation scores for the eight fine-scale behaviors.*

**Scores**	**Appetitive Exploratory**	**Biting**	**Burst Swimming**	**Feeding**	**Fleeing**	**Nibbling**	**Passing**	**Startle Response**
Precision	0.54	0.17	1.00	0	0.25	1.00	0.63	1.00
Recall	0.54	0.11	1.00	0	0.14	0.33	0.83	0.38
F1	0.54	0.13	1.00	0	0.18	0.50	0.71	0.55
Balanced Accuracy	0.71	0.51	1.00	0.43	0.55	0.67	0.82	0.69
Average	0.58	0.23	1.00	0.11	0.28	0.63	0.75	0.65
